# Supplementary material for: Mechanical forces and ligand binding modulate Pseudomonas aeruginosa PilY1 mechanosensitive protein
Source: Life Sci Alliance. 2025 Mar 7;8(5):e202403111. doi: 10.26508/lsa.202403111 (PMC11891296; doi:10.26508/lsa.202403111)
Supplement: Supplementary file 1 [file LSA-2024-03111_TableS1.docx]

| **Intermediate** | **Unfolding force, pN (mean±SEM)**  **EGTA** | **# events**  **EGTA** | **Unfolding force, pN (mean±SEM)**  **Ca^2+^** | **# events**  **Ca^2+^** | **Significance (*p<0.05, **p<0.01,**  **X p>0.05)** |
| --- | --- | --- | --- | --- | --- |
| **I1** | 11.8±0.5 | 141 | 9.4±0.3 | 172 | ** |
| **I2**  **I2A**  **I2B** | 17.9±0.7  19.5±0.9  19.6±0.9 | 101  40  40 | 15.7±0.6  16.8±0.7  16.8±0.6 | 113  59  59 | *  *  * |
| **Variable ints.** | 27.0±0.2 | 541 | 29.4±0.2 | 611 | ** |
| **I3** | 26.5±0.4 | 140 | 25.8±0.4 | 172 | X |
| **I4** | 26.9±0.4 | 140 | 30.4±0.4 | 172 | ** |
| **I5** | 27.0±0.4 | 132 | 30.9±0.4 | 157 | ** |
| **I6** | 27.2±0.5 | 97 | 30.9±0.6 | 86 | ** |
| **I7** | 27.5±1.0 | 27 | 32.2±1.3 | 19 | * |
| **I8** | 32.0±3.0 | 5 | 32.1±1.7 | 3 | X |
| **I9** | N/A | N/A | 32.3 | 1 | N/A |
| **Last int.** | 27.8±0.4 | 141 | 30.8±0.4 | 172 | ** |
