# Supplementary material for: Mechanical forces and ligand binding modulate Pseudomonas aeruginosa PilY1 mechanosensitive protein
Source: Life Sci Alliance. 2025 Mar 7;8(5):e202403111. doi: 10.26508/lsa.202403111 (PMC11891296; doi:10.26508/lsa.202403111)
Supplement: Supplementary file 3 [file LSA-2024-03111_TableS3.docx]

| **Intermed.** | **I1** | **I2/I2A/I2B** | **I3** | **I4** | **I5** | **I6** | **I7** | **I8** | **Last int.** |
| --- | --- | --- | --- | --- | --- | --- | --- | --- | --- |
| **I1** |  | **  **  ** | ** | ** | ** | ** | ** | ** | ** |
| **I2**  **I2A**  **2B** |  |  | **  **  ** | **  **  ** | **  **  ** | **  **  ** | **  **  ** | **  **  ** | **  **  ** |
| **I3** |  |  |  | ** | ** | ** | ** | ** | ** |
| **I4** |  |  |  |  | X | X | X | X | X |
| **I5** |  |  |  |  |  | X | X | X | X |
| **I6** |  |  |  |  |  |  | X | X | X |
| **I7** |  |  |  |  |  |  |  | X | X |
| **I8** |  |  |  |  |  |  |  |  | X |
| **Last int.** |  |  |  |  |  |  |  |  |  |
