# Supplementary material for: Mechanical forces and ligand binding modulate Pseudomonas aeruginosa PilY1 mechanosensitive protein
Source: Life Sci Alliance. 2025 Mar 7;8(5):e202403111. doi: 10.26508/lsa.202403111 (PMC11891296; doi:10.26508/lsa.202403111)
Supplement: Supplementary file 4 [file LSA-2024-03111_TableS4.docx]

| **Intermed.** | ***k_U_^0^* (s^-1^)**  **EGTA** | ***∆x* (nm)**  **EGTA** | ***k_U_^0^* (s^-1^)**  **Ca^2+^** | ***∆x* (nm)**  **Ca^2+^** |
| --- | --- | --- | --- | --- |
| **I3** | (5.9 ± 2.7) ×10^-3^ | 0.35 ± 0.06 | (2.3 ± 0.9) ×10^-3^ | 0.51 ± 0.05 |
| **I4** | (3.5 ± 2.5) ×10^-2^ | 0.30 ± 0.10 | (2.3 ± 1.0) ×10^-3^ | 0.42 ± 0.05 |
| **I5** | (1.6 ± 0.9) ×10^-2^ | 0.62 ± 0.07 | (6.6 ± 7.7) ×10^-2^ | 0.39 ± 0.15 |
| **I6** | (1.3 ± 0.8) ×10^-1^ | 0.43 ± 0.08 | (9.4 ± 1.1) ×10^-1^ | 0.43 ± 0.08 |
